# Draft Genome Sequence of Lacticaseibacillus rhamnosus IMI 507023

**DOI:** 10.1128/mra.01217-21

**Published:** 2022-04-07

**Authors:** Ivana Nikodinoska, Jenny Makkonen, Daniel Blande, Colm Moran

**Affiliations:** a Alltech European Headquarters, Sarney, Dunboyne, County Meath, Ireland; b Biosafe – Biological Safety Solutions Ltd., Kuopio, Finland; c Regulatory Affairs Department, Alltech SARL, Vire, France; SIPBS, University of Strathclyde

## Abstract

We report here the draft genome sequence of Lacticaseibacillus rhamnosus strain IMI 507023, a lactic acid bacterium, isolated from corn silage in Nicholasville, Kentucky, USA. The strain is deposited in the Centre for Agriculture and Bioscience International (CABI) Culture Collection with the accession number IMI 507023.

## ANNOUNCEMENT

Lactic acid bacteria (LAB) have a versatile metabolism, and antimicrobial production improves fermented foods and feed production (1, 2). *Lacticaseibacillus* may promote successful forage fermentation, leading to improved feed hygiene and increased nutrient availability to livestock (2–4). Here, we describe the draft genome sequence of Lacticaseibacillus rhamnosus IMI 507023.

A 10-g sample of silage, obtained from a farm located in Kentucky, was homogenized with 90 mL of 0.85% NaCl solution, plated onto de Man-Rogosa-Sharpe (MRS) agar, and incubated anaerobically at 37°C for 48 h for colony isolation. For genomic analyses, the isolates were grown in 10 mL of MRS broth and incubated anaerobically at 37°C for 16 to 17 h. Genomic DNA was extracted according to the sample preparation and lysis protocol described for Gram-negative and some Gram-positive bacterial samples in the Qiagen Genomic DNA Handbook and purified according to Genomic-tip 100/G (Qiagen) instructions. DNA was submitted to Eurofins Genomics (Constance, Germany) for library preparation (NEBNext Ultra II FS DNA library prep kit; New England Biolabs, San Diego, CA, USA) and whole-genome sequencing (NovaSeq 6000; Illumina), producing 2 × 150-bp paired-end reads. The sequencing produced 6,106,545 raw reads. The reads were trimmed using Trimmomatic v0.38.1 (5) and assembled using Unicycler v0.4.8 (6), generating 39 contigs with a genome length of 2,880,392 bp, 608-fold coverage, GC content of 46.75%, and an *N*_50_ value of 141,355 bp. The annotation with the NCBI Prokaryotic Genome Annotation Pipeline (PGAP) v6.0 (7) predicted 2,565 coding genes, 48 tRNA genes, and 2 rRNA genes. The IMI 507023 genome showed a Mash (8) (MinHash software v0.1.1) distance of 0.001041 and a calculated OrthoANI (9) (OrthoANI software v1.40) value of 99.98% with L. rhamnosus DSM 20021 (GenBank accession number BALT00000000).

Searches for antimicrobial resistance genes were made against the NCBI Bacterial Antimicrobial Resistance Reference Gene Database (NCBI BioProject number PRJNA313047; database version 2021-06-01.1), using AMRFinderPlus v3.10.5 (10) and DIAMOND (11) (Galaxy v0.9.29.0). Searches were also performed against the ResFinder database (12) (downloaded on 20 April 2021) using blastn (BLAST+ v2.12.0). Default parameters were used except where otherwise stated. No acquired antimicrobial resistance genes of concern that were above 80% identity and 70% coverage (13) were found.

Toxin and virulence factor genes potentially carried by IMI 507023 were screened using DIAMOND v2.0.8 (settings, mode blastp –more-sensitive –matrix ‘BLOSUM62’ –comp-based-stats ‘1’ –masking ‘1’ –evalue ‘0.01’ –id ‘80’ –query-cover ‘0’ –subject-cover ‘0’) against a virulence factor database (VFDB; accessed on 14 July 2021) (14, 15). No genes encoding potential virulence or pathogenicity factors were identified.

Plasmids were searched from the genome data by screening the contigs against the PlasmidFinder database (accessed on 14 July 2021) (16). Assembly files were examined for circular contigs, and BLAST (BLAST+ v2.12.0) searches were conducted to determine if contigs were likely to be plasmids. The analysis was performed using ABRicate v1.0.1 (https://github.com/tseemann/abricate) with settings of minimum identity 50% and minimum coverage 50%. No plasmid-related sequences matched against the PlasmidFinder database.

### Data availability.

All data are available under BioProject accession number PRJNA771511. The raw reads were deposited at the SRA under accession number SRR17939987. The GenBank accession number is JAJBZL000000000.
